# The microbiological diagnostic performance of metagenomic next-generation sequencing in patients with sepsis

**DOI:** 10.1186/s12879-021-06934-7

**Published:** 2021-12-16

**Authors:** Di Ren, Chao Ren, Renqi Yao, Lin Zhang, Xiaomin Liang, Guiyun Li, Jiaze Wang, Xinke Meng, Jia Liu, Yu Ye, Haoli Li, Sha Wen, Yanhong Chen, Dan Zhou, Xisi He, Xiaohong Li, Kai Lai, Ying Li, Shuiqing Gui

**Affiliations:** 1 Department of Critical Care Medicine, Shenzhen Second People’s Hospital, The First Affiliated Hospital of Shenzhen University, Shenzhen, China; 2 Trauma Research Center, Fourth Medical Center of the Chinese PLA General Hospital, Beijing, 100048 People’s Republic of China; 3 Research & Development, Dinfectome Inc., Nanjing, 213164 Jiangsu China; 4 Department of Neurosurgery, Shenzhen Longgang Central Hospital (The Second Affiliated Hospital of the Chinese University of Hong Kong (Shenzhen)), Shenzhen, China

**Keywords:** Metagenomic next-generation sequencing, Microbial culture, Intensive care units, Sepsis, Pathogens

## Abstract

**Background:**

In this study, we aimed to perform a comprehensive analysis on the metagenomic next-generation sequencing for the etiological diagnosis of septic patients, and further to establish optimal read values for detecting common pathogens.

**Methods:**

In this single-center retrospective study, septic patients who underwent pathogen detection by both microbial culture and metagenomic next-generation sequencing in the intensive care unit of the Second People’s Hospital of Shenzhen from June 24, 2015, to October 20, 2019, were included.

**Results:**

A total of 193 patients with 305 detected specimens were included in the final analysis. The results of metagenomic next-generation sequencing showed significantly higher positive rates in samples from disparate loci, including blood, bronchoalveolar lavage fluid, and cerebrospinal fluid, as well as in the determination of various pathogens. The optimal diagnostic reads were 2893, 1825.5, and 892.5 for *Acinetobacter baumannii, Pseudomonas aeruginosa*, and *Klebsiella pneumoniae*, respectively.

**Conclusions:**

The metagenomic next-generation sequencing is capable of identifying multiple pathogens in specimens from septic patients, and shows significantly higher positive rates than culture-based diagnostics. The optimal diagnostic reads for frequently detected microbes might be useful for the clinical application of metagenomic next-generation sequencing in terms of timely and accurately determining etiological pathogens for suspected and confirmed cases of sepsis due to well-performed data interpretation.

## Background

Sepsis is a characteristic of multiple organ dysfunction due to an uncontrolled host response to pathogens and is one of the leading causes of mortality in intensive care units (ICUs) [1, 2]. Vincent et al. [3] conducted an international audit of ICU patients worldwide and found that 29.5% of critically ill patients developed sepsis on admission or during the ICU stay, accounting for 25.8% of deaths. In China, the incidence of sepsis was reported to be 20.6% among patients who received ICU care, with an overall 90-day mortality of 35.5%, but it reached 51.94% when complicated by septic shock, posing a great threat to clinical prognosis [4]. The prompt and accurate identification of pathogens is indeed essential for the clinical management and outcome improvement of septic patients. It has been demonstrated that delayed initiation of antimicrobial treatments is closely associated with higher mortality rates of septic patients, which appears to be more prominent with prolonged administration [5]. Currently, culture-based diagnostic procedures are deemed the golden standard for detecting bacteremia but present limited information due to low positive rates, false results due to contamination and over administration of antibiotics, hindering their usefulness, especially for critically ill patients [6].

Metagenomic next-generation sequencing (mNGS) is widely used for detecting environmental microorganisms and is a highly efficient tool for diagnosing bacterial sepsis [7, 8]. A study by Long et al. [9] showed that the diagnostic sensitivity of pathogens in septic patients was significantly increased by next-generation sequencing when compared to blood culture. Dai and colleagues found that mNGS was capable of identifying etiological agents when the results of blood culture were negative owing to post-antibiotic treatment [10]. However, standard practices of mNGS for diagnosing specific microorganisms among critically ill patients remain scarce. In this study, we performed a comprehensive analysis on the application of mNGS for the etiological diagnosis of septic patients admitted to the ICU, and further established optimal read values for determining common pathogens by making comparisons with the results of routine microbial culture. We present the following article in accordance with the STARD reporting checklist.

## Methods

### Study population and sample collection

This single-center retrospective study was conducted in the ICU department of the Second People’s Hospital of Shenzhen, a tertiary care hospital located in Shenzhen, China. All ICU patients diagnosed with sepsis who underwent pathogen detection via both microbial culture and mNGS from June 24, 2015, to October 20, 2019, were incorporated into the current study. The diagnostic criteria of sepsis were in accordance with the Surviving Sepsis Campaign: International Guidelines for Management of Sepsis and Septic Shock: 2016 (Sepsis 3.0): suspected or confirmed infection plus the value of Sequential Organ Failure Assessment (SOFA) ≥ 2 within 24 h after ICU admission. Specimens from the blood, bronchoalveolar lavage fluid (BALF), cerebrospinal fluid (CSF), and urine of these patients were collected within 24 h of sepsis onset. Repeated tests were also conducted due to potential secondary infection based on the results of routine blood tests. Samples were collected and further subjected to pathogen detection by both regular microbial culture and the mNGS method. Oral or written consent was obtained from all patients, and this study was complied with the Declaration of Helsinki and was approved by the Ethics Committee of the Second People’s Hospital of Shenzhen (KS20190521004-FS2019052906).

### Data collection

Data were extracted from the electronic patient record system of the Second People’s Hospital of Shenzhen by using predesigned data collection forms. Demographic characteristics (age and sex), comorbidities, laboratory findings (routine blood test, C-reactive protein [CRP] and procalcitonin [PCT] levels), sources of infection, and clinical interventions (antibiotic usage, emergency surgery, tracheal intubation, mechanical ventilation, red blood cell transfusion, and renal replacement therapy) were collected. Data from prognostic scoring systems, including the SOFA and Acute Physiology and Chronic Health Evaluation II (APACHE II), were also obtained. We chose all-cause ICU mortality as the prognostic indicator of the present study.

### Metagenomic next-generation sequencing and analysis

#### DNA extraction

DNA from different samples was extracted by using the TIANamp Micro DNA Kit (DP316, Tiangen Biotech, Beijing, China) following the manufacturer’s operational manual. The DNA was dissolved in tris-ethylenediaminetetraacetic acid buffer and further used for construction of the DNA libraries. The quantity and quality of DNA was assessed using the Qubit (Thermo Fisher Scientific) and NanoDrop (Thermo Fisher Scientific), respectively.

#### Construction of DNA libraries and sequencing

The extracted DNA was used to generate 200–300 bp fragments by a Bioruptor Pico device. The DNA libraries were constructed through DNA-fragmentation, end-repair, adapter-ligation, and polymerase chain reaction (PCR) amplification. An Agilent 2100 Bioanalyzer (Agilent Technologies, Santa Clara, CA) was used for quality control of the DNA libraries. Quality qualified libraries were subsequently sequenced on the BGISEQ-50 platform, and at least 20M reads were obtained for each sample. The samples from healthy volunteers were used as negative controls that underwent the same process with clinical samples.

#### Bioinformatic analysis

We use in-house developed bioinformatics pipeline for pathogen identification. Briefly, high-quality sequencing data were generated by removing low-quality and short (length < 35 bp) reads, followed by computational subtraction of human host sequences that were mapped to the human reference genome (hg19) by using Burrows–Wheeler alignment. The remaining data were classified by simultaneous alignment to four microbial genome databases consisting of viruses, bacteria, fungi, and parasites. The classification reference databases were downloaded from NCBI (ftp://ftp.ncbi.nlm.nih.gov/genomes/). RefSeq contained 4061 whole genome sequences of viral taxa, 2473 bacterial genomes or scaffolds, 199 fungi, and 135 parasites, which were associated with human diseases.

#### Interpretation and reporting

We used the following criteria for positive results of mNGS:

1. For Mycobacterium and Legionella pneumophila, the result was considered positive if a species detected by mNGS had the reads per million (RPM) ≥ 1.
2. For bacteria (excluding Mycobacterium and Legionella pneumophila), virus with significantly different from the human genome sequence (such as Adenovirus, Influenza virus), the result was considered positive if a species detected by mNGS had the RPM ≥ 3.
3. For RPM of fungi ≥ 5, RPM of parasites ≥ 10, the result was considered positive if a species was detected by mNGS.

### Statistical analysis

Demographic characteristics of all enrolled patients were summarized and presented as the mean (standard deviation [SD]), median (interquartile range [IQR]), and count (proportion) when appropriate. The positive ratios between mNGS and blood culture among disparate type of samples were compared by applying the McNemar test and Fisher’s exact test as appropriate. Receiver operating characteristic (ROC) curve analysis was performed to assess the diagnostic efficiency of mNGS in comparison with conventional microbiological methodology and to confirm the optimal reads for frequently detected pathogens. The optimal cut-off values of reads were determined in line with the maximum Youden index at this point. In addition, two tailed *P* values less than 0.05 were deemed statistically significant. The aforementioned statistical analyses were conducted by using IBM SPSS 19.0 software.

## Results

### Baseline characteristics and clinical features

A total of 193 patients were diagnosed with sepsis and included in the current study. The median age of the enrolled patients was 57.7 years (IQR, 15–96), and 71 (36.8%) patients were female (Table 1). The most common source of infection was the lungs (151 [78.2%]), followed by the urinary tract (32 [16.6%]), skin and soft tissue (12 [6.2%]), peritoneal cavity (9 [4.7%]), central nervous system (9 [4.7%]), and biliary tract (5 [2.6%]). Comorbidities were also commonly noted in septic patients, such as anemia (103 [53.4%]), hypertension (76 [39.4%]), diabetes (43 [22.3%]), chronic cardiac dysfunction (33 [17.1%]), and chronic renal dysfunction (25 [13.0%]). The median SOFA and APACHE II scores were 9 (IQR, 7–11) and 19 (IQR, 13–25), respectively. All septic patients received antibiotic treatments, and other advanced measures were administered as organ supports, including mechanical ventilation (151 [78.2%]), infusion of red blood cells (138 [71.5%]), renal replacement therapy (91 [47.2%]), and even emergency surgery (23 [11.90%]). Overall, 57 (29.5%) septic patients died during hospitalization.

**Table 1:** Demographic characteristics and outcomes of included patients (n=193)

Characteristics	
Age, years median (IQR)	57.7 (15–96)
Gender, female, n (%)	71 (36.8)
Source of infection	
Lungs, n (%)	151 (78.2)
Peritoneal cavity, n (%)	9 (4.7)
Biliary tract, n (%)	5 (2.6)
Urinary tract, n (%)	32 (16.6)
Skin and soft tissue, n (%)	12 (6.2)
Central nervous system, n (%)	9 (4.7)
Others, n (%)	3 (1.6)
Laboratory tests	
White blood cells, 10^9^/L, median (IQR)	10.6 (7.0–17.1)
Total amount of lymphocytes, 10^9^/L, median (IQR)	0.84 (0.5–1.3)
Total amount of neutrophils, 10^9^/L, median (IQR)	9.0 (5.5–14.8)
Ratio of lymphocytes, (%), median (IQR)	4.8 (0.17–10.5)
Ratio of neutrophils, (%), median (IQR)	77.8 (0.94–88.75)
CRP	79.58 (28.7–156.18)
PCT	2.14 (0.47–7)
Blood glucose	8.3 (6.45–11.85)
Blood lactate	2.1 (1.5–3.1)
Comorbidities	
Chronic cardiac dysfunction, n (%)	33 (17.1)
Diabetes, n (%)	43 (22.3)
Chronic respiratory disease, n (%)	22 (11.4)
Chronic renal dysfunction, n (%)	25 (13.0)
Hepatic cirrhosis, n (%)	1 (0.5)
Anemia, n (%)	103 (53.4)
Trauma, n (%)	27 (14.0)
Hypertension, n (%)	76 (39.4)
Prognostic scoring systems	
SOFA, median (IQR)	9 (7–11)
APACHE II, median (IQR)	19 (13–25)
Interventions	
Antibiotics, n (%)	193 (100%)
Emergency surgery, n (%)	23 (11.90)
Tracheal intubation, n (%)	129 (66.8)
Mechanical ventilation, n (%)	151 (78.2)
Infusion of red blood cells, n (%)	138 (71.5)
Renal replacement therapy, n (%)	91 (47.2)
Outcome	
Mortality, n (%)	57 (29.5)

*IQR* interquartile range, *CRP* C-reactive protein, *PCT* procalcitonin, *SOFA* sequential organ failure assessment, *APACHE II* Acute Physiology and Chronic Health Evaluation II

### Comparison between the culture-based diagnostic procedure and mNGS

**Fig. 1:**
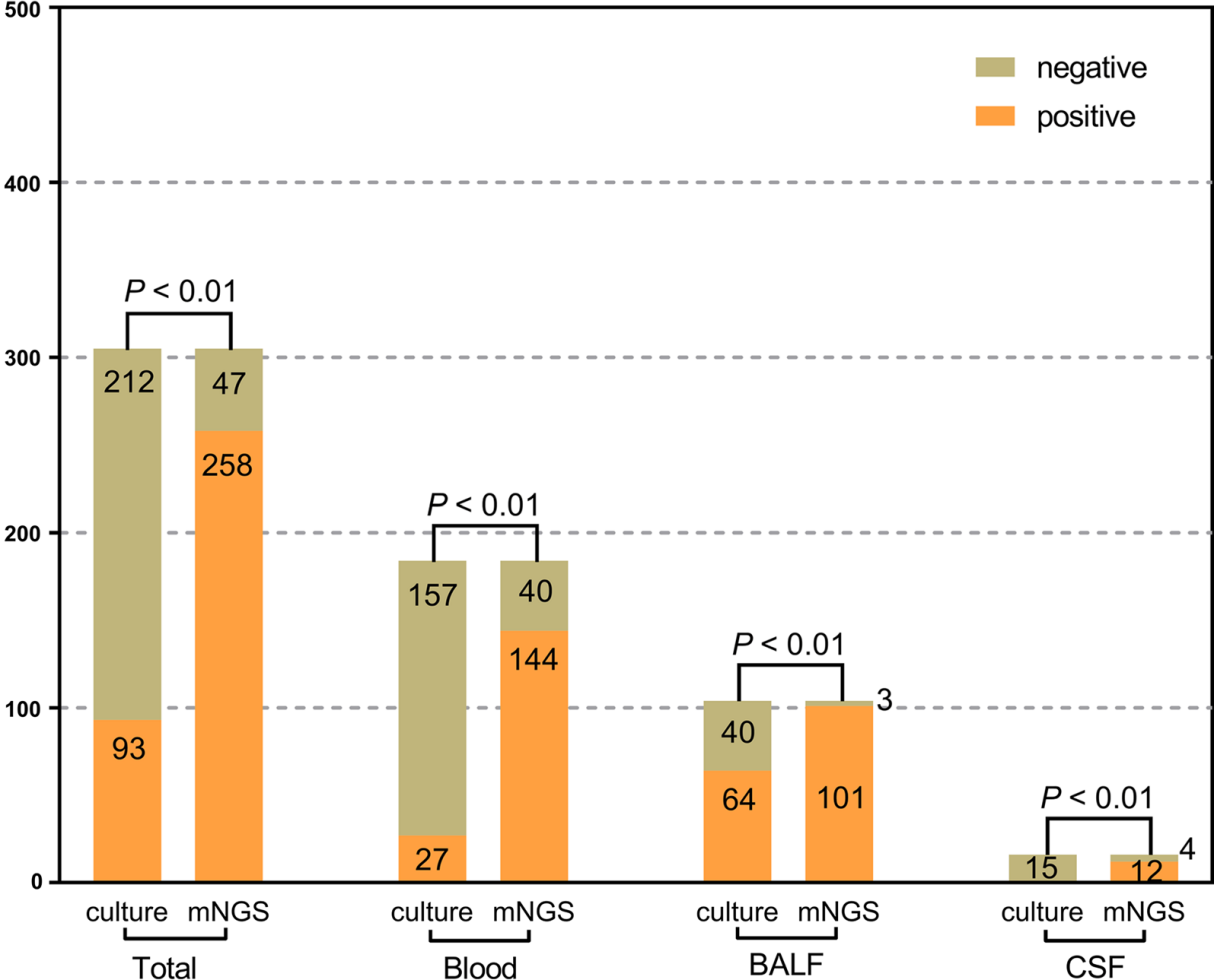
The positivity of disparate sample types between metagenomic next-generation sequencing (mNGS) and microbial culture. Among all detected samples, the positive rates of mNGS were significantly higher than those of culture. A similar tendency was observed in all types of specimens, including blood, bronchoalveolar lavage fluid (BALF) and cerebrospinal fluid (CSF). A *P* value of McNemar test or Fisher’s exact test lower than 0.05 was deemed as statistically significant

**Fig. 2:**
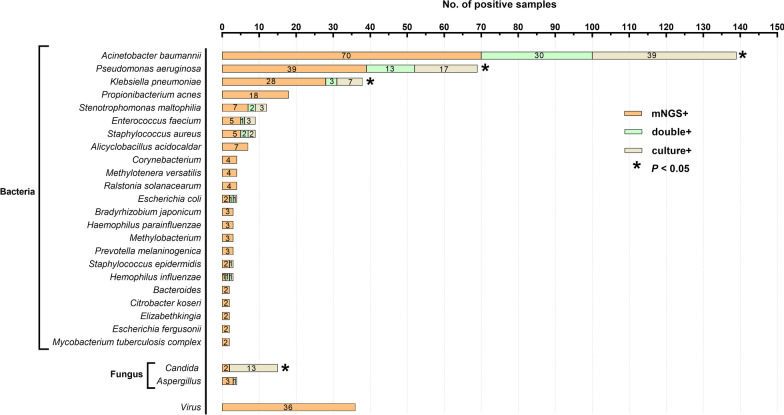
The positivity of disparate pathogenic microorganisms between metagenomic next-generation sequencing (mNGS) and microbial culture. *Acinetobacter baumannii*, *Pseudomonas aeruginosa* and *Klebsiella pneumoniae* were the most commonly isolated bacteria from septic specimens, which were also found to be significantly more detectable with mNGS than with conventional culture. Interestingly, the mNGS method demonstrated obviously lower positive rates than culture-based diagnostics in terms of Candida detection. Viral infection was solely detected with mNGS. A *P* value of McNemar test or Fisher’s exact test lower than 0.05 was deemed as statistically significant

In total, 305 samples were collected and underwent both culture-based diagnostics and mNGS: 184 (60.3%) blood specimens, 104 (34.1%) BALF specimens, 16 (5.2%) CSF specimens, and 1 (0.3%) urine specimen (Fig. 1). Among all detected samples, the positive rate of mNGS was 84.6%, which was significantly higher than that of culture-based diagnostics (mNGS vs. culture: 84.6% vs. 30.5%, *P *< 0.01). A consistent tendency was also observed in distinct specimens: blood (mNGS vs. culture: 78.3% vs. 14.7%, *P *< 0.01), BALF (mNGS vs. culture: 97.1% vs. 61.5%, *P *< 0.01), and CSF (mNGS vs. culture: 75% vs. 6.3%, *P *< 0.01). We further conducted a comparison between mNGS and culture-based diagnostics for detecting different microbes and found that *Acinetobacter baumannii* (139 [45.6%]) was the most common isolated bacteria in septic patients in our study, followed by *Pseudomonas aeruginosa* (69 [22.6%]), *Klebsiella pneumoniae* (38 [12.5%]), and *Propionibacterium acnes* (18 [5.9%]), which showed higher positive rates with mNGS than with culture-based diagnostics (Fig. 2). Furthermore, fungi, including *Candida* and *Aspergillus*, were detected by both mNGS and culture-based diagnostics. Of note, the mNGS method demonstrated obviously lower positive rates than culture-based diagnostics in terms of *Candida* detection. A total of 36 (11.8%) samples were noted to have viral infection, which was solely identified by mNGS.

**Fig. 3:**
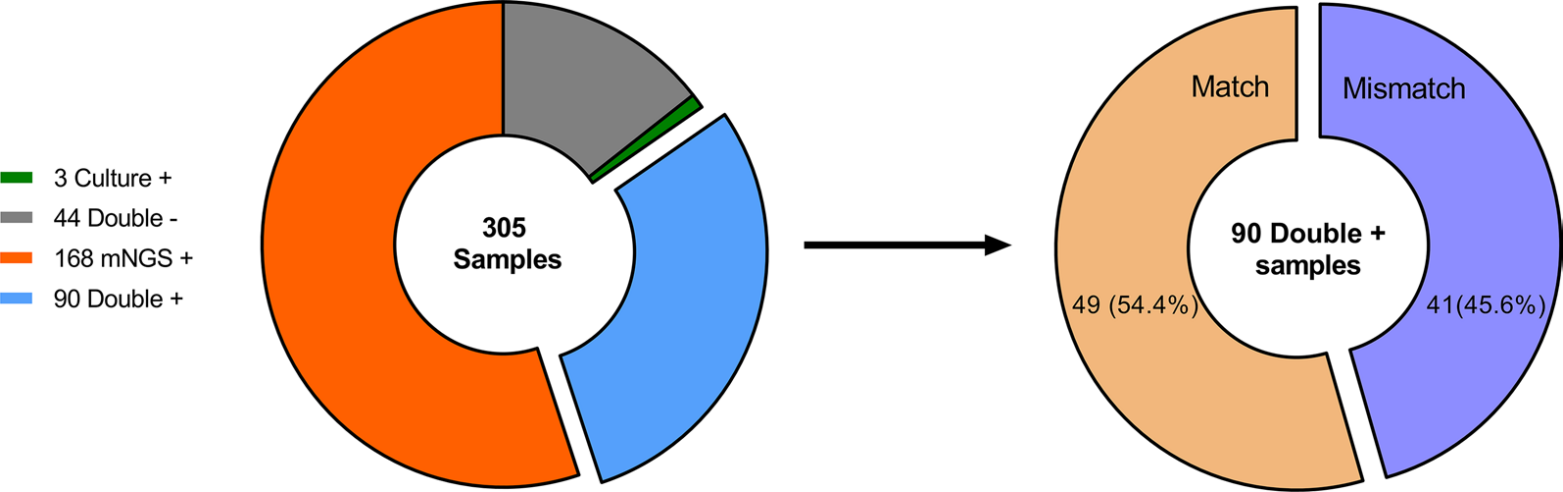
Concordance analysis between metagenomic next-generation sequencing (mNGS) and culture. Culture and mNGS showed double positive results in 90 (29.5%) specimens, in which 49 (54.4%) cases were completely matched, while mismatch was observed in 41 (45.6%) cases

In this study, culture and mNGS measures demonstrated double positive results in 90 (29.5%) specimens and double-negative results in 44 (14.4%) specimens, while 168 (55.1%) samples and 3 (1.0%) samples had positive results only with mNGS or culture alone, respectively. Among the specimens that had positive results from both methods, 49 (54.4%) were completely matched, while mismatch was observed in 41 (45.6%) cases (Fig. 3).

### The optimal reads for common pathogens

**Fig. 4:**
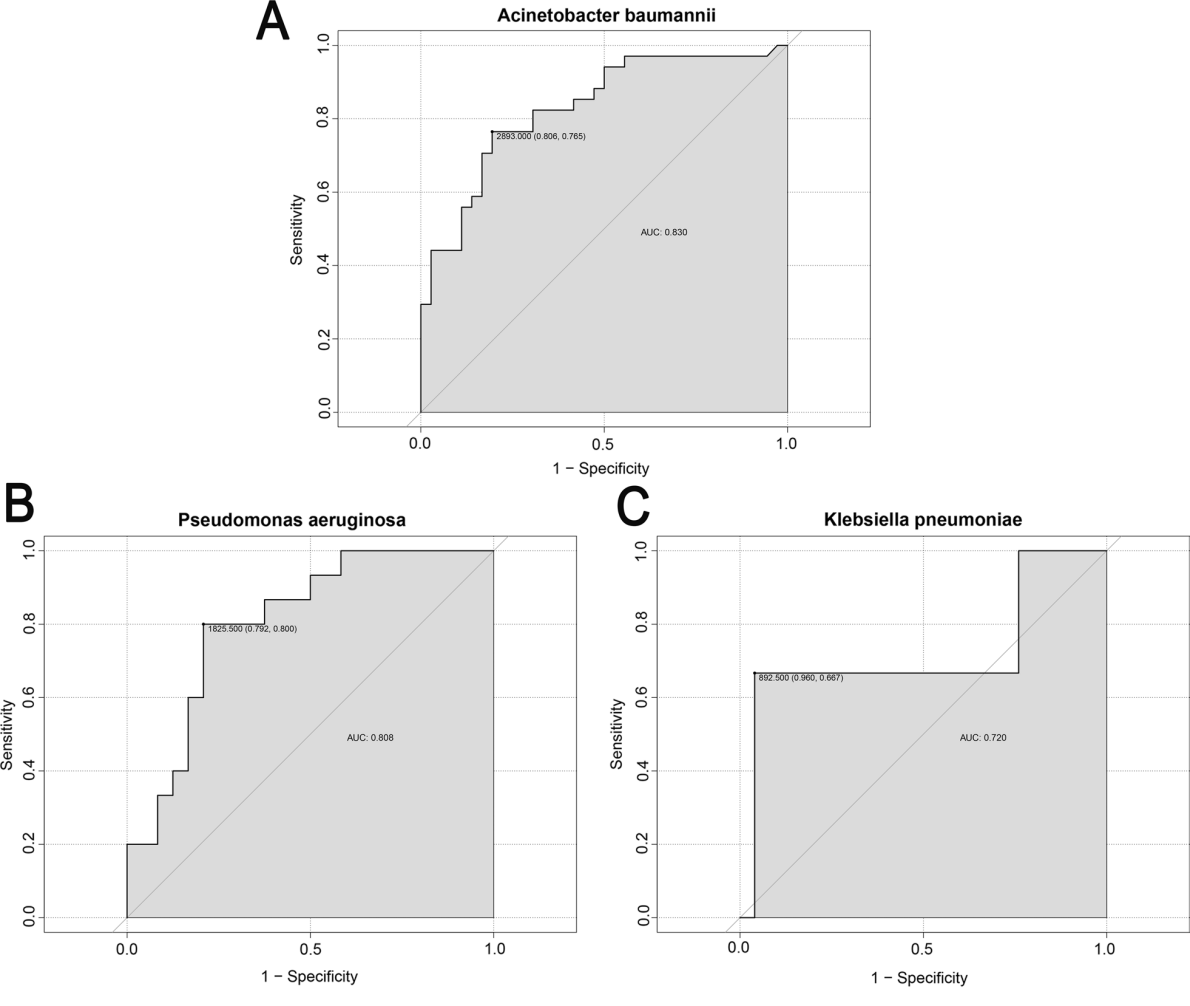
The optimal reads for commonly detected pathogens. The receiver operating characteristic (ROC) curve analysis was performed for confirming the optimal reads for frequently detected pathogens. The optimal cut-off values of reads were determined in line with the maximum of Youden index at this point. **A** *Acinetobacter baumannii*; **B** *Pseudomonas aeruginosa*; **C** *Klebsiella pneumoniae*

Given the importance of read value in the interpretation of mNGS results, we applied ROC curve analysis for determining the optimal read values for diagnosing the most frequently detected bacteria in our center. The optimal diagnostic reads were 2893 (specificity: 0.806, sensitivity: 0.765), 1825.5 (specificity: 0.792, sensitivity: 0.8), and 892.5 (specificity: 0.96, sensitivity: 0.667) for *Acinetobacter baumannii, Pseudomonas aeruginosa*, and *Klebsiella pneumoniae*, respectively, and demonstrated relatively high sensitivity and specificity (Fig. 4). The area under the curve (AUC) values revealed an acceptable performance of the reads for detecting these bacteria: 0.83 for *Acinetobacter baumannii*, 0.808 for *Pseudomonas aeruginosa*, and 0.73 for *Klebsiella pneumoniae.*

## Discussion

mNGS is broadly applied for detecting pathogens and especially for the timely and accurate diagnosis of critical illness due to suspected etiology microbes, such as sepsis, a severe condition that brings about poor outcomes for ICU patients. Herein, we conducted a comprehensive analysis on the diagnostic performance of mNGS for detecting pathogens among septic patients in comparison with routine culture-based diagnostics. We found that elderly patients were more commonly complicated by sepsis in the ICU, and the lungs were the major source of infection that caused sepsis, which might be partly attributed to anatomical features and age-associated comorbidities, including hypertension, diabetes, chronic cardiac dysfunction, and chronic respiratory disease. All septic patients received empirical antibiotic treatments at ICU admission mainly based on signs of infection in routine blood tests, including elevated counts in white blood cells and neutrophils, and increased levels of blood CRP and PCT. Indeed, these septic patients in our study presented with severe conditions, as evidenced by high SOFA and APACHE II scores, and needed further advanced treatments, including mechanical ventilation, infusion of red blood cells, and renal replacement therapy. Of note, septic patients had a high ICU mortality rate, sharing the same clinical characteristics with severe sepsis patients from a previous report by Xie et al. [4].

In the current study, mNGS showed obviously higher positive rates of pathogen detection than culture-based diagnostics in all samples as well as in various types of specimens, such as blood, BALF, and CSF. For example, the positive rate of blood culture was merely 14.7%, which was much lower than that of mNGS (78.3%) for detecting pathogens with systematic exposure. This was also applied to determine local infection, as noted, with higher positive rates in both BALF and CSF samples, indicating the general applicability of mNGS for pathogenic detection, even in samples with relatively low positive rates by culture-based diagnostic procedures. These benefits were noted in previously reported studies, which suggested that mNGS exerted a valuable diagnostic platform for determining relevant pathogens [11]. We further compared the diagnostic performance between mNGS and culture-based diagnostic procedures for isolating different kinds of microbes. mNGS was capable of identifying various pathogens with negative results by culture-based diagnostics and showed higher positive rates in common pathogens for the development of sepsis, including *Acinetobacter baumannii*, *Pseudomonas aeruginosa*, and *Klebsiella pneumoniae*. These commonly identified pathogens in this subset of patients conformed with previously published reports on etiological microorganisms for septic patients in ICUs, suggesting that Gram-negative organisms were the main cause for the development of in-hospital sepsis [4, 12]. In fact, the positive rates of culture-based diagnostics were less than 50% of that by mNGS, which was mainly due to the administration of empiric antibiotics. However, a study by Grumaz and colleagues [11] revealed that the divergent distribution of pathogen infection in postoperative septic patients by mNGS, such as *Escherichia coli*, *Enterococcus faecium*, and *Bacteroides fragilis*, was partly due to different sources of septic patients as well as sample types. For the results showed that culture positive but NGS negative in some patients, maybe due to the load of pathogens is under the detection threshold. Moreover, the noteworthy double-positive rates between mNGS and culture-based diagnostics shed light on the fairly good diagnostic performance of mNGS. In addition, for the mismatch results with double samples, we would introduce a third-party detection method for verification, and will be shown in the subsequent study. Remarkably, 19 of those 33 patients showed mNGS-guided clinical responses.

The early identification of fungal infection is of clinical significance with the use of mNGS and has been confirmed by many previous studies [9, 13]. In this study, *Candida* and *Aspergillus* were the major fungi isolated by both mNGS and culture-based diagnostics. However, the positive rates of fungus identification with mNGS were markedly lower than those with culture-based diagnostics, which showed similar results compared to previous studies that reported low-positive rates in fungi detection by mNGS in septic patients [9, 11]. In addition, a total of 36 samples were found to have viral infection by mNGS, indicating extensive pathogenic information for clinical practice by mNGS in septic patients. It has been demonstrated that reactivation of latent viruses is frequently complicated in prolonged sepsis and is critically involved in the progression and outcome of septic patients [14].

The application of mNGS is mainly restricted to identifying clinically relevant pathogens based on data from coverage, depth, and reads. Currently, the read values of mNGS are commonly used for the interpretation of distinct pathogenic infections after optimization [15, 16]. However, cut-off reads for diagnosing distinct microbes by mNGS and their clinical applications in septic patients remain unclarified. In this study, we applied ROC analysis to determine the optimal cut-off read values for the three most commonly detected pathogens based on the results of culture-based diagnostics. The optimal cut-off read values for these bacteria were relatively high, from 892.5 to 2893, and showed acceptable sensitivity and specificity. To our knowledge, this is the first report to identify the cut-off reads for diagnosing distinct pathogens, especially for patients with sepsis, which is indeed favorable for the clinical application of mNGS. In fact, mNGS has been used to isolate distinct microbes in various diseases, such as *Streptococcus pneumoniae* in pediatric bacterial meningitis, *Ebola* virus disease, and arthritis caused by *Legionella micdadei* as well as *Staphylococcus aureus *[15, 17, 18]. Although these reports show good performance in terms of pathogen detection, few provide optimal cut-off reads for each pathogen. The definite cut-off reads for pathogens indeed facilitate the extensive application and optimize the data interpretation of mNGS.

Nevertheless, some issues should be taken into consideration when interpreting our results. First, this study was conducted by means of retrospective analysis, which limited comprehensive data analysis and further information on the use of antibiotics. Recently, we registered and performed a prospective study for the evaluation of mNGS in pathogen detection and antibiotic administration in septic patients from the ICU. Second, a relationship between the read values and prognoses of septic patients was absent in this observation due to the relatively small sample size of patients with distinct pathogen infections, which requires further investigation. Third, the mNGS was capable of exporting data on mixed infections of multiple microbes, especially for the reactivation of fungi and viruses, which were pivotal factors for the prognostic assessment of septic patients and should be considered in further studies with large sample sizes.

## Conclusions

mNGS is capable of identifying multiple pathogens in disparate types of samples from septic patients, and shows higher positive rates than culture-based diagnostics. The optimal read values for distinct microbes might be useful for the clinical application of mNGS in term of timely and accurately determining the etiology of pathogens in sepsis due to well-performed data interpretation.

## Data Availability

All data generated or analyzed during this study are included in this published article. The data of this study are available from the corresponding author on reasonable request.

## Abbreviations

AUC: Area under the curve
APACHE II: Acute physiology and chronic health evaluation II
BALF: Bronchoalveolar lavage fluid
CRP: C-reactive protein
CSF: Cerebrospinal fluid
ICU: Intensive care unit
IQR: Interquartile range
mNGS: Metagenomic next-generation sequencing
PCT: Procalcitonin
PCR: Polymerase chain reaction
ROC: Receiver operating characteristic
SD: Standard deviation
SOFA: Sequential organ failure assessment
